# Telehealth for Pediatric Gastroenterology Care Now: The Transition to Telehealth and the Impact of Webinar-Based Didactics

**DOI:** 10.1097/PG9.0000000000000182

**Published:** 2022-04-08

**Authors:** Mala Setty, Edward B. Mougey, Elizabeth Berg, John M. Rosen, Jennifer Lee, B.U.K. Li, Rajitha D. Venkatesh, James P. Franciosi

**Affiliations:** From the * Department of Pediatrics, UCSF Benioff Children’s Hospital, Oakland, CA; † Center for Pharmacogenomics and Translational Research, Nemours Children’s Health System, Jacksonville, FL; ‡ Department of Pediatrics, Columbia University Irving Medical Center, New York City, NY; § Division of Pediatric Gastroenterology, Pediatric Intestinal Rehabilitation Center, Hepatology and Nutrition, NewYork-Presbyterian Morgan Stanley Children’s Hospital, New York City, NY; ∥ Division of Pediatric Gastroenterology, Hepatology, and Nutrition, Children’s Mercy Kansas City, Kansas City, MO; ¶ Division of Pediatric Gastroenterology, Hepatology, and Nutrition, Nationwide Children’s Hospital, Columbus, OH; # Department of Pediatrics, Medical College of Wisconsin, Milwaukee, WI; ** Division of Gastroenterology, Hepatology and Nutrition, Nemours Children’s Hospital, Orlando, FL; †† Department of Pediatrics, University of Central Florida College of Medicine, Orlando, FL.

**Keywords:** telehealth, webinar, didactics, pediatrics, gastroenterology

## Abstract

**Objectives:**

With the coronavirus disease 2019 public health emergency (PHE), telehealth (TH) became essential for continued delivery of care. Members of the North American Society for Pediatric Gastroenterology, Hepatology and Nutrition (NASPGHAN) formed the Telehealth for Pediatric Gastrointestinal Care Now (TPGCN) working group and rapidly organized a telemedicine webinar to provide education and guidance. We aim to describe the webinar development and prospectively assess the effectiveness of this webinar-based educational intervention.

**Methods:**

NASPGHAN members who registered for the TPGCN webinar received pre- and post-webinar surveys. Outcome measures included a modified Telehealth Acceptance Model (TAM) survey and a Student Evaluation of Educational Quality (SEEQ) standardized instrument.

**Results:**

Seven hundred seventy-six NASPGHAN members participated in the webinar, 147 (33%) completed the pre-webinar survey; of these, 25 of 147 (17%) completed a post-webinar survey. Before the PHE, 50.3% of the pre-webinar survey participants had no TH knowledge. Webinar participants trended to have increased acceptance of TH for follow-up visits (pre-webinar, 68% versus post-webinar, 81%; *P* = 0.15) and chronic disease care (pre-webinar, 57% vs post-webinar, 81%; *P* = 0.01). The overall acceptance of TH as shown by TAM pre-webinar was 1.74 ± 0.8, which improved to 1.62 ± 0.8 post-webinar (lower scores indicate greater acceptance; *P* < 0.001). SEEQ results indicate that webinar material was understandable (post-webinar, 95%). Participants found breakout sessions informative and enjoyable (post-webinar, 91%).

**Conclusion:**

The TPGCN TH webinar was an effective educational intervention that fostered increased TH usage for follow-up and chronic care visits, improved TAM scores, and was well received by participants as seen by high SEEQ scores. Sustained and expanded pediatric gastrointestinal TH usage beyond the coronavirus disease 2019 PHE is expected.

**What Is Known**

- Telehealth (TH) grew rapidly in response to the coronavirus disease 2019 (COVID-19) public health emergency (PHE).
- The COVID-19 PHE disrupted professional medical education.

**What Is New**

- Of the gastrointestinal providers, 50.3% had no experience with TH before the COVID-19 PHE.
- Measures of the Telehealth Acceptance Model indicate increasing acceptance of TH and recognition of its advantages and potential limitations.
- Acceptance of TH grew for direct patient care, particularly in chronic disease management, and E visits and interprofessional consultations were identified as areas with growth potential.
- This study adds to the literature on the effectiveness of virtual training in healthcare and highlights the importance of recognizing the needs of learners.

Before the coronavirus disease 2019 (COVID-19) public health emergency (PHE) on March 13, 2020, deployment of telehealth (TH) in pediatric gastroenterology and broadly throughout healthcare in the United States was limited with disparate adoption due to regional regulatory, insurance, provider, and healthcare system barriers.^1^ Since inception of the COVID-19 PHE, hospital and healthcare entities worldwide have abruptly pivoted from face-to-face patient encounters to TH encounters to reduce exposure and spread of the highly contagious severe acute respiratory syndrome coronavirus 2.

Most providers had no experience with TH before the COVID-19 PHE.^2^ Consequently, the PHE established an urgent need for provider-focused TH education. Initial published TH guidance for pediatric gastroenterologists was published in “Coronavirus Disease 2019 and the Pediatric Gastroenterologist,” which stated “the use of telemedicine is now a critical tool”^3^ and specific best practice considerations were included in “COVID-19-A Guide to Rapid Implementation of [Pediatric gastrointestinal (GI)] Telehealth Services.”^4^

In response to the evolving needs and interests of the members of the North American Society for Pediatric Gastroenterology, Hepatology and Nutrition (NASPGHAN), the Telehealth for Pediatric GI Care Now (TPGCN) ad hoc working group was formed to rapidly organize a timely TH webinar to provide clinician education, practice models of care, and guidance for documentation and reimbursement. The primary focus of this article is to describe the rapid development of and effectiveness of a TH webinar for the pediatric gastroenterology providers in response to the COVID-19 PHE. The secondary aim was to understand knowledge, attitudes, and practice habits related to TH.

## METHODS

### Development of TPGCN

During March 2020, as the COVID-19 PHE unfolded, in accordance with the US Centers for Disease Control and Health and Humans Services guidance, typical in-person pediatric gastroenterology clinic visits and elective procedures were largely curtailed. With little experience and no road map, private practices, medical centers, and hospitals in the United States began to rapidly ramp up TH in as little as 2-week time. Based on the needs of the pediatric GI community, B.U.K.L. and J.P.F. rapidly organized the ad hoc TPGCN working group of NASPGHAN. The initial purpose of TPGCN was to develop a comprehensive telemedicine webinar to provide guidance to those pediatric GI clinicians who were actively implementing telemedicine in their practice. This working group was composed of 23 physician members and representatives from 6 pertinent committees including Clinical Practice, Electronic Health Record (special interest group), Clinical Care and Quality, Training, Professional Education, and Technology. Over a 6-week period, TPGCN planned, funded, and conducted 2 live online educational sessions (June 10 and 17, 2020).

### Pre-NASPGHAN TH Webinar Planning and Content Development

Appropriate webinar topics were identified using a modified Delphi method and were subsequently developed, reviewed, and finalized into the curriculum (See Supplemental Digital Content Table 1, http://links.lww.com/PG9/A73). The webinar focused on 5 content areas: (1) technical aspects (video platforms, information technology training, troubleshooting); (2) documentation, billing, and reimbursement (consent and Health Insurance Portability and Accountability Act of 1996 compliance, electronic health record integration, physical exam, billing, cross-state licensure); (3) practice management (scheduling, pre-visit orientation, indications for telemedicine versus in-person visits, multidisciplinary visits, patient satisfaction); (4) inpatient care (virtual rounding, teleconsultation); and (5) tele-education (conferences, virtual fellow and resident supervision).

The format was finalized with feedback from a webinar technical expert and the NASPGHAN council. The structure featured prerecorded (to avoid connectivity issues) 30-minute didactic sessions (topics 1, 2, and 3) followed by 4 interactive breakouts (topics 1, 2, 3, and 4/5) designed with a multimodal format combining didactic slides, case-based discussion, interactive polls, and open question and answer (Q&A). The webinar totaled 3 hours of prerecorded didactics and 4 hours of live breakout sessions. There was a rolling registration, and participants could choose to participate in some or all of the live sessions.

All 23 members of the TPGCN working group served as didactic presenters, moderators, discussants, or some combination of the three. Practice sessions for didactic and breakout sessions were conducted to enhance effective delivery and smooth transitions. Each live (synchronous) breakout session was semistructured to allow a robust Q&A using online chat discussion while providing salient prepared take-home messages. To facilitate access to the Q&A during later on-demand (asynchronous) use, 10 pediatric gastroenterology fellows edited breakout session transcripts into an online-accessible document organized by subtopic. All didactic sessions and breakout transcripts remain accessible to NASPGHAN members at the time of this publication.

### Study Population

Registration for the TPGCN webinar, both live and on demand, was available to all NASPGHAN members including physicians, nurse practitioners, dietitians, psychologists, and gastroenterology fellow trainees. Informed consent was obtained from all participants before participating in the surveys. As of October 20, 2020, a self-selected group of 776 members participated in the study. Of the 776 total participants, 294 registered for the live TH webinar, of whom 147 completed the pre-webinar survey (pre-webinar, n = 147/294; 50%) and 37 completed the 3-month post-webinar survey following completion of either the live or on-demand webinar (post-webinar, n = 37/776; 4.8%; See Supplemental Digital Content Figure 1, http://links.lww.com/PG9/A73). Of the 37 participants who completed the post-webinar survey, 25 also completed the pre-webinar survey (paired pre/post-webinar group, n=25/37; 68%; See Supplemental Digital Content Figure 2, http://links.lww.com/PG9/A73). Details about participant characteristics and training are given in Supplemental Digital Content Table 2, http://links.lww.com/PG9/A73.

### TPGCN Webinar Assessments

Participants were surveyed up to three times; one pre- and two different post-webinar surveys. Pre- and post-webinar surveys were administered 2 days before and 3 months after the webinar. Only participants who registered before the live webinar were eligible for the pre-webinar survey. All participants (live or on demand) were eligible for the 3-month post-webinar survey. In addition, all participants who completed the webinar received an immediate post-webinar assessment for feedback and evidence of mastery of the course. Surveys are reproduced in Supplemental Digital Content Appendix 1, http://links.lww.com/PG9/A73.

The pre- and post-webinar surveys were developed to include questions about demographics, knowledge of TH, current practice habits, and overall outlook toward TH. Knowledge and practice habit questions were developed in agreement with the research group. Portions of the previously validated Telehealth Acceptance Model^5^ (TAM) survey questions were used to gauge overall outlook toward TH. The TAM survey is a 7-point Likert scale survey, and it is the most widely used model to measure acceptance of technology among healthcare providers.^6^ It incorporates factors of acceptance predicted by attitudes toward the use, perceived usefulness, and perceived ease of use of technology (individual value judgments).^5,7^ Specific domains focus on attitudes towards use, shaped by self-efficacy, perceived ease of use of technology, perceived usefulness, and perceived incentives, which are individual value judgments.^5,7^

The immediate post-webinar survey included the learning composite portion of the Student Evaluation of Educational Quality (SEEQ) questionnaire survey.^8^ The SEEQ is a previously validated and reliable student feedback questionnaire. It has an exceptionally high level of reliability (r^2^ = 0.88 to 0.97) and correlates significantly with a wide range of measures of learning outcomes including student feelings of mastery of course content and plans to apply learned skills.^8^

### Statistical Analysis

Survey data were preprocessed by dropping not applicable responses and by collapsing data for Likert items from 5 categories down to 3 categories. For example, strongly agree and agree were combined into one category labeled agree. Collapsed categories for non-Likert survey questions are indicated by / in the tables where they appear. Where appropriate, Likert items that were originally coded from unfavorable to favorable were recoded from favorable to unfavorable so that responses for all questions in a given analysis were coded in the favorable-to-unfavorable direction. Analyses were conducted in R base, version 4.0.2 (2020).^9^ Comparison of proportions for baseline characteristics between groups (count data) was performed using a 2-sided Fisher exact test (exact *P* value). A 2-sided Wilcoxon test (exact *P* value) was used for comparison of distributions. For TAM analysis, survey questions were assigned to TAM domains as indicated in Supplemental Digital Content Appendix 1, http://links.lww.com/PG9/A73.^5,10^

## RESULTS

As of October 20, 2020, there were a total of 776 registrants, 294 of whom registered for the live webinar. The highest attended session was the Practice Management session with 101 live and 313 on-demand attendees (414 total). The second highest attended session was Documentation and Billing with 65 live attendees and 321 on-demand attendees (386 total). The live attendances for the breakout discussion sessions were 60.7% for the Technical breakout, 71.4% for the Billing/Documentation breakout, 57.1% for the Practice Management breakout, and 53.6% for the Inpatient and Tele-Education breakout. The pre-webinar survey was completed by 147 of the 294 registrants (pre-webinar group, 50%). A total of 37 participants (37/776, 4.8%) completed the 3-month post-webinar survey (post-webinar group). Twenty-five participants (25/37, 68%) completed both pre- and 3-month post-webinar surveys (paired pre/post-webinar group).

**FIGURE 1.**
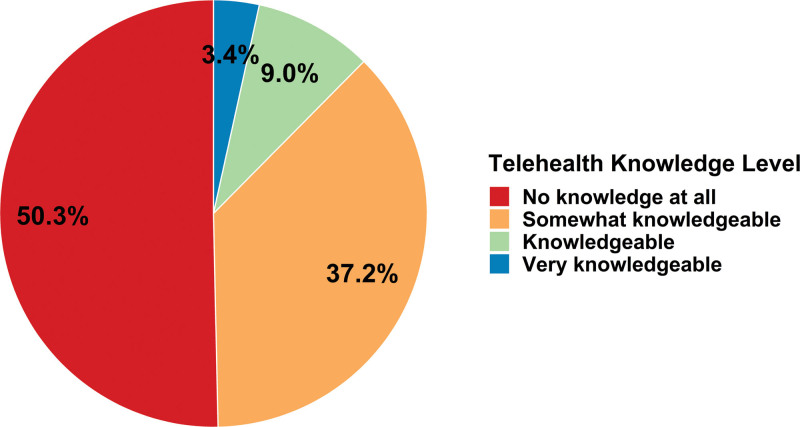
Self-assessed pre-COVID-19 public health emergency telehealth knowledge level of participants who completed the per-webinar survey (147 respondents).

Among the 147 participants who completed the pre-webinar survey, 58.5% were female (n = 86), and the age distribution was 20% 20 to 30 years of age (n = 30), 34.7% 36 to 50 years of age (n = 51), and 42.9% >50 years of age (n = 63). Participant practice type was 56.5% academic hospital-based practice (n = 83), 15.6% community hospital-based practice (n = 23) and 27.9% mixed/other (n = 41). Occupations of participants included 78.2% MD/DO (n = 115), 7.48% advanced registered nurse practitioner/nurse practitioner (n = 11), and 14.3% dietitian/other (n = 21). Pre-COVID knowledge of TH and previous telemedicine training are shown in Figure 1. Before the COVID-19 PHE, 50.3% (73/145) of the pre-webinar respondents reported no knowledge of TH. Only 8.2% (12/147) reported no knowledge of TH during the PHE.

**FIGURE 2.**
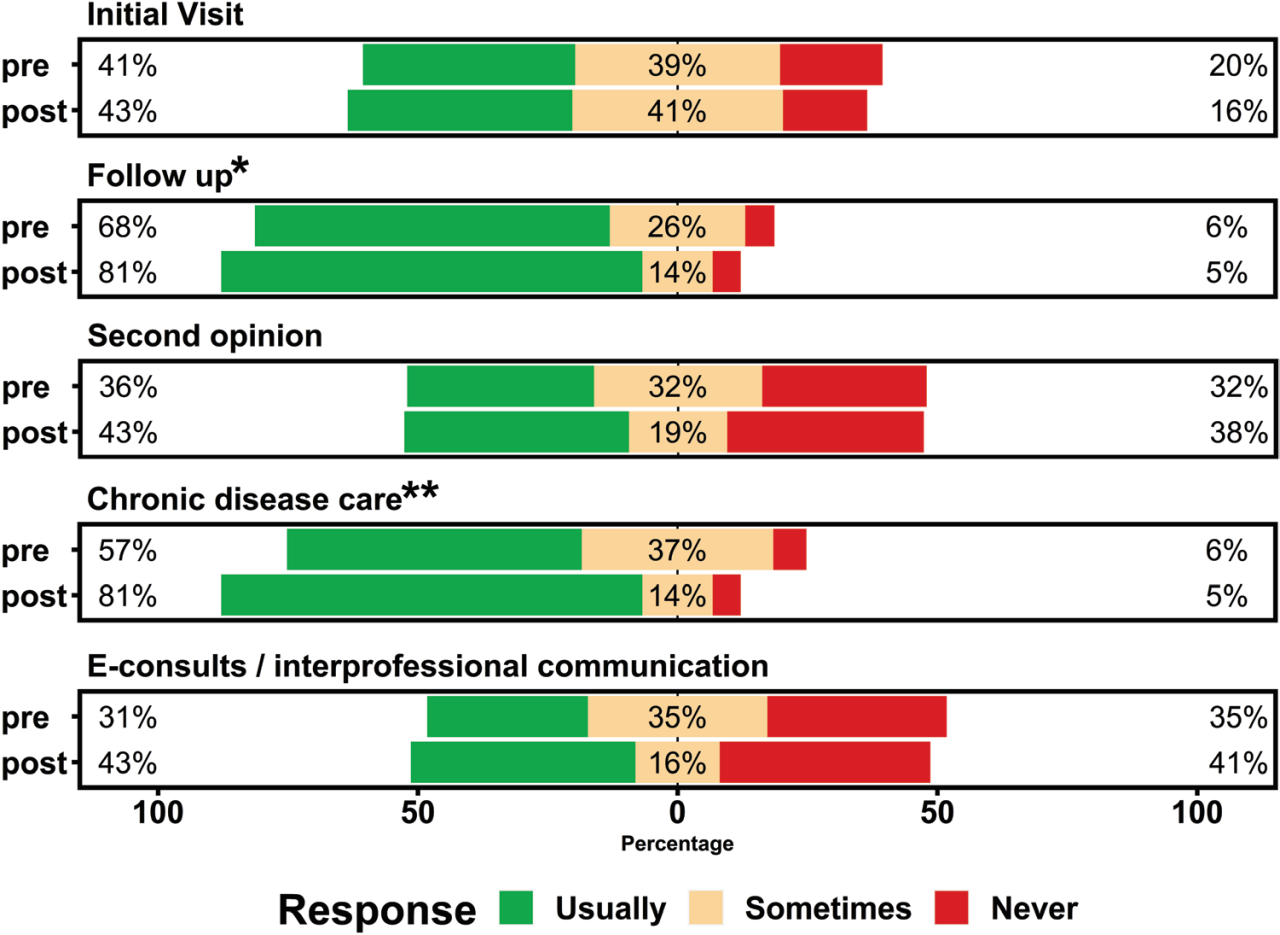
Practice habits of telehealth, pre- and post-webinar. Preferences for utilization of telehealth for various visit types showed that in general, respondents were significantly more likely to use telehealth for ongoing management of patients with chronic disease. There was also a trend toward a preference to utilize telehealth for follow-up visits rather than for initial visits (including second opinions) and e-consults or interprofessional communications. Responses that exhibit a trend (*P* ≤ 0.1) or differ significantly (*P* ≤ 0.05) between groups by Wilcoxon analysis are indicated by *P ≤ 0.1 and **P ≤ 0.05.

The results of the practice habit questions, which assessed the types of visits conducted via TH, are shown in Figure 2. Following the webinar participants most often used and improved their use of TH for follow-up visits (pre-webinar, 68% versus post-webinar, 81%; *P* = 0.15) and chronic disease care (pre-webinar, 57% versus post-webinar, 81%; *P* = 0.01). Most participants were not using TH for new patients (pre-webinar, 25% versus post-webinar, 22%), second opinions (pre-webinar, 36% versus post-webinar, 43%) or E-consultation (pre-webinar, 31% versus post-webinar, 43%).

The TAM scores for the pre-webinar group compared to the post-webinar group are summarized in Table 1. The overall acceptance of TH, as measured by TAM scores, improved from pre-webinar to post-webinar (1.74 ± 0.8 to 1.62 ± 0.8, *P* < 0.001; lower scores indicate greater acceptance). The highest levels of acceptance, regardless of webinar participation, were seen in the accessibility of patients measures (pre-webinar, 1.17 ± 0.4 versus post-webinar, 1.11 ± 0.4; *P* = 0.31). All domains of TAM showed greater agreement after the webinar with self-efficacy (pre-webinar, 1.61 ± 0.75 versus post-webinar, 1.32 ± 0.60; *P* < 0.01), perceived usefulness (pre-webinar, 2.00 ± 0.84 versus post-webinar, 1.87 ± 0.84; *P* = 0.04), and perceived ease of use (pre-webinar, 1.50 ± 0.68 versus post-webinar, 1.31 ± 0.64; *P* = 0.01) improving significantly. The specific question with the greatest pre- and post-webinar change was “I have rich experiences on telemedicine” (pre-webinar, 1.98 ± 0.8; post-webinar, 1.51 ± 0.7; *P*< 0.01). Domains: Accessibility of Medical Records, Perceived Incentives, Attitude Toward Use, and Accessibility of Patients were essentially unchanged following the webinar, while domain Behavioral Intention to Use, which is a function of all other domains combined, trended towards greater acceptance, but the improvement did not reach significance (pre-webinar, 1.52 ± 0.71; post-webinar, 1.38 ± 0.64; *P* = 0.27).

**TABLE 1.** Telehealth Acceptance Model analysis

Domain*	Mean (SD)		*P* value†
	Pre	Post	Post vs pre
SE aggregate	1.61 (0.75)	1.32 (0.60)	<0.01‡
Most visits I do can be accomplished by video visits	1.45 (0.68)	1.24 (0.55)	0.07
I have rich experiences on telemedicine	1.98 (0.79)	1.51 (0.69)	<0.01‡
I can use the equipment properly	1.41 (0.63)	1.19 (0.52)	0.02‡
AMR aggregate	1.42 (0.58)	1.39 (0.55)	0.85
I can gather the correct information and easily record a patient’s health condition into the EMR	1.36 (0.54)	1.39 (0.55)	0.71
Because of the precise record of the patients, it enables me to provide appropriate care for my patients	1.48 (0.61)	1.40 (0.55)	0.55
AP aggregate	1.17 (0.43)	1.11 (0.36)	0.31
With telehealth, I can be in contact with patients who seldom come to the clinic	1.20 (0.47)	1.17 (0.45)	0.71
With telehealth, I can be in contact with patients who have transportation difficulties	1.14 (0.38)	1.06 (0.23)	0.26
PEU aggregate	1.50 (0.68)	1.31 (0.64)	0.01‡
It is easy to learn to use the software and equipment for telemedicine	1.42 (0.63)	1.19 (0.52)	0.02‡
It is easy to perform my job with the EMR and telemedicine software	1.57 (0.73)	1.43 (0.73)	0.21
PU aggregate	2.00 (0.84)	1.87 (0.84)	0.04‡
Telemedicine will positively affect patient quality of care and treatment plans	1.30 (0.58)	1.17 (0.45)	0.18
I can conduct a thorough patient exam using telemedicine	2.35 (0.79)	2.03 (0.81)	0.03‡
Video visits are more acceptable for established patients rather than new patients	2.61 (0.64)	2.69 (0.62)	0.38
I believe that my patients will receive better care in person than via telehealth	2.33 (0.73)	2.17 (0.74)	0.22
Telehealth is efficient for diagnosing patients and scheduling	1.60 (0.71)	1.47 (0.70)	0.29
Telehealth makes it possible to provide more comprehensive care service	1.84 (0.80)	1.69 (0.75)	0.34
PI aggregate	1.93 (0.84)	1.90 (0.87)	0.66
Current Medicare/Medicaid reimbursement during the Public Health Epidemic is adequate	1.83 (0.73)	1.85 (0.76)	0.89
I would like the temporary expansion of telehealth to become permanent	1.22 (0.48)	1.19 (0.46)	0.72
I feel concern regarding liability issues with telemedicine	2.33 (0.77)	2.24 (0.86)	0.67
I am concerned about state medical licensure issues with telemedicine	2.36 (0.76)	2.32 (0.85)	0.99
ATU aggregate	1.99 (0.86)	1.92 (0.90)	0.54
Most visits I do can be accomplished by video visits	1.64 (0.80)	1.61 (0.84)	0.75
I feel that there is a loss of personal contact with patients that results from telemedicine	2.34 (0.78)	2.22 (0.87)	0.52
BIU aggregate	1.52 (0.71)	1.38 (0.64)	0.27
I will care for more of my patients using telemedicine in my practice	1.52 (0.71)	1.38 (0.64)	0.27

ATU = Attitude Toward Use; BIU = Behavioral Intention to Use; EMR = electronic medical record; PEU = Perceived Ease of Use; PI = Perceived Incentives; PU = Perceived Usefulness; SE = Self-Efficacy.

*Level key: 1 = agree, 2 = neither agree nor disagree, 3 = disagree; lower scores indicate greater acceptance. Levels from the original survey have been condensed.

†A Wilcoxon test was used for comparison of distributions.

‡Distributions are significantly different between groups.

**FIGURE 3.**
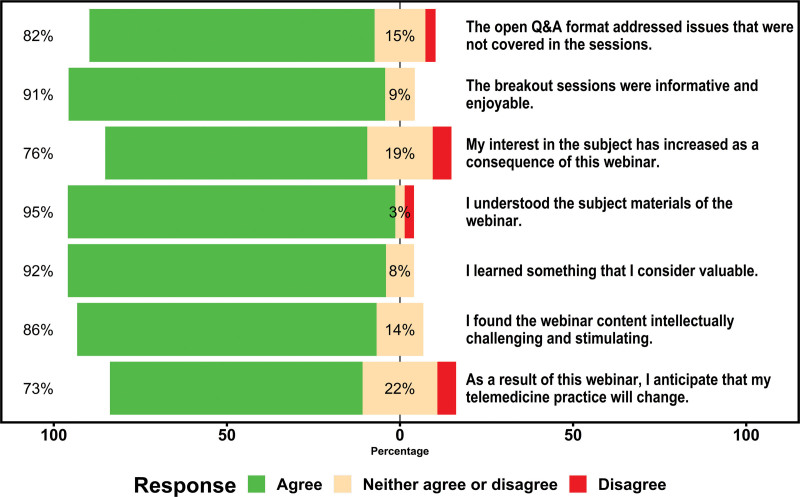
The Students’ Evaluations of Educational Quality (SEEQ) is an instrument for collecting a participant’s evaluation of the webinar. The SEEQ measures distinct components of the webinar’s effectiveness. Q&A = question and answer.

Results of the SEEQ (Fig. 3) indicate the material was presented in an understandable way (post-webinar, 95%) and participants found breakout sessions informative and enjoyable (post-webinar, 91%). The results of SEEQ indicate a high level of satisfaction with the webinar educational intervention and learning format. In their feedback, 62.8% of post-webinar participants anticipated that their telemedicine practice would change as a result of the webinar, 67.4% of participants felt the interactive Q&A format of the breakout sessions addressed issues that were not covered in the didactics, and 90.7% of participants stated they would attend similar webinar-based formats with live interactive learning.^11^

## DISCUSSION

During the COVID-19 PHE, rapid TH implementation presented unique challenges for providers due to limited prior experience and variable support within practices. Challenges including weak internet connectivity, TH platform hardware and software security, virtual practice operations, multidisciplinary care integration, billing and coding adaptations, interstate licensure, and academic integration of trainees12 have been described. The TPGCN webinar aimed to support NASPGHAN members in the rapid transition from in-person care to TH. A group of 23 volunteer physicians developed and executed a highly attended webinar within 2 months of declaration of the PHE. Based on low prior knowledge and experience reported among participants, there was a clearly identified and timely need for education at the beginning of the COVID-19 PHE.

The TPGCN webinar format was designed to fit adult learners by providing live and on-demand viewing. A number of participants registered for the webinar before the live (synchronous) date, and many more opted to view it asynchronously (83.2% of attendees for session 1 and 75.6% for session 2). The high subscription to on-demand (asynchronous) attendance was unanticipated and differed from previously published literature on adult learners’ opinions about synchronous versus asynchronous learning.^11^ The immediate post-webinar survey results indicated over 90% valued the live interactive format of the breakout sessions and would attend a similar live webinar again.

Webinars and web conferencing in professional training and education have become increasingly critical during the PHE and have exhibited efficacy in delivering gains in knowledge and skills. When enabled with modern infrastructure and software, these technological tools can provide effective means for students and professional learners in educational progress. Virtual learning has the bimodal benefit of live didactics (synchronous) and prerecorded on-demand lectures (asynchronous) suitable to the needs of the individual learner. A study from the Accreditation Council for Graduate Medical Education comparing synchronous to asynchronous learning found approximately 90% of learners preferred synchronous learning.^11^ In another example, virtual hospital ward rounds among a small sample of 14 medical students were well-received with over 92% recommending this form of teaching.^13^

With this recent shift in teaching methods, studies examining the efficacy and impact of virtual medical and professional education have gained more traction. Assessment of acquired knowledge and measures of learning quality are essential to determining that learning objectives can be met. The SEEQ subscore on learning provided positive feedback on the educational quality of the webinar evidenced by an overall high level of satisfaction. Participants indicated a high level of knowledge acquisition and intention to put this knowledge into practice. Cited barriers to successful learning include confidentiality and security concerns (i.e., Zoom-bombing),^12^ loss of face-to-face teaching, hardships in maintaining focus and concentration,^14^ and potential for ineffective learning strategies, poor motivation, and lack of feedback. Alternatively, professional adult trainees often actively research online during virtual presentations to enlighten and provoke additional higher-level discussions.

Advances in communications, digital streaming technology, and user interfaces^15^ have allowed for ease of access potentially reaching across the globe and reducing geographic and economic barriers.^16^ Online higher education research has demonstrated that a sense of community is essential for effective learning and increased satisfaction, absent in the asynchronous formats.^14^ We theorize that this particular downside may have been tolerated during the PHE given geographic constraints impacting time zones and logistics related to increased demands on clinicians during this time. This may also reflect the demand for this topic matter was so high that learners overlooked the preference for synchronous learning in order to obtain TH training in whatever format was feasible.

Several domains in our TAM survey regarding acceptance of TH improved over time. Providers indicated they could have rich experiences with TH, and trends indicated an increased level of comfort with using the technology when queried several months into the transition. Additionally, an enhanced comfort with the equipment and software could be attributed to the effect of the webinar intervention and amplified usage of TH at that time. There was general agreement that TH improved patient access and accessibility of subspecialty care. Our results suggest a high utilization of TH for specific instances in clinical care, such as follow-up visits, particularly in chronic disease management, and indicated increased use over time.

TAM results also identified certain perceived limitations that impacted acceptance of TH technology such as concerns regarding an insufficient physical examination, potential for inadequate quality of care in certain cases, and barriers regarding insurance regulations and licensure. The underutilization of TH technology was noted for initial consultations, second opinion visits, as well as E-consults and interprofessional communications. This exposes gaps that may be ripe for transformation and suggests targets for practice modifications and future technologies to overcome these potential deficiencies. The ethics and liability concerns for medical practice via TH will be scrutinized in a forthcoming position statement from NASPGHAN.

Our present study has several limitations. The demographics and characteristics portion of the survey was only given to participants in the pre-webinar group, which limited our ability to confirm that the pre- and post-webinar groups were well matched. The survey may have measured the change in TH acceptance over this period of time as practitioners’ experience grew using TH rather than the effects of the webinar alone being measured. The webinar was held during a rapidly evolving early period when colleagues were more cautious, less comfortable using TH, and, therefore, may not be generalizable to other subject matter. Though the response rates fit the typical average external survey response rate of 10% to 30%,^17^ the limited number of participants who completed both pre- and post-webinar surveys reduced our power in paired comparison where each participant served as their own control.

In conclusion, the TPGCN TH webinar was a successful and effective educational intervention in response to the rapid need to convert practices from in-person to TH visits. As a result of the webinar, there were increases in follow-up and chronic care TH usage. Improved Self-Efficacy, Perceived Ease of Use, and Perceived Utility aggregate scores following the webinar suggest that the intervention may have increased acceptance of telemedicine within our membership.

The next phase of development in educational initiatives on TH can focus on how best to sustain and evolve TH practices beyond the PHE.

## Supplementary Material


