# Telehealth for Pediatric Gastroenterology Care Now: The Transition to Telehealth and the Impact of Webinar-Based Didactics: Erratum

**DOI:** 10.1097/PG9.0000000000000361

**Published:** 2023-08-22

In the following article published in *JPGN Reports*, the sixth author’s middle initial was erroneously omitted from the final publication: Rajitha D. Venkatesh.
