# Supplementary material for: Telehealth for Pediatric Gastroenterology Care Now: The Transition to Telehealth and the Impact of Webinar-Based Didactics
Source: JPGN Rep. 2022 Apr 8;3(2):e182. doi: 10.1097/PG9.0000000000000182 (PMC10158463; doi:10.1097/PG9.0000000000000182)
Supplement: Supplementary file 1 [file pg9-3-e182-s001.pdf]

Telehealth for Pediatric Gastroenterology Care Now: The Transition to Telehealth and the Impact of Webinar-based Didactics

Online Appendix Supplement

Appendix 1a: Pre-Webinar Survey

A. Pre-Test Questions

1. Which of the following are acceptable ways to bill for a telehealth visit?

   1 Standard components including a physical examination and medical decision making
   2 Face-to-face time based billing
   3 Total visit time base billing including pre-visit planning and post-visit care.
   4 Any of the above may be acceptable depending on the policies of your institution/practice

2. Which of the following are acceptable components of a telehealth visual physical examination?

   1 No hepatosplenomegaly
   2 No palpable masses appreciated
   3 Abdomen non-distended
   4 All of the above

1. The CPT GT telehealth modifier is a standard for all telehealth visits regardless of insurance type.

   1 True
   2 False

3. Obtaining informed consent before conducting telemedicine (telehealth, telephone and e-visits) is:

   1 A requirement in many but not all states
   2 A condition of reimbursement by some insurance providers
   3 A and B
   4 None of the above

4. Three weeks after discharge from an inpatient hospitalization, a patient's father contacts his GI provider via the electronic health record secure patient portal. He asks a question about the appearance of his child's gastrostomy tube (GT) site and includes a photo of the GT site in the message. The GI provider reviews the image the next day and responds with recommendations for management of granulation tissue. The GI provider documents the care provided and charges for the visit as a:

   1 Telehealth visit
   2 Virtual Check-in
   3 E-visit
   4 Telephone visit

B. Demographics

5. Age?
   1 20-35 years

2 36-50 years
3 51-65 years
4 65+ years
5 Prefer not to answer

6. How do you identify?

1 Male
2 Female
3 Non-binary/third gender
4 Prefer not to answer
5 Prefer to self describe (please specify)

7. Type of practice?

1 Academic hospital based practice
2 Community hospital based practice
3 Mixed
4 Other

8. Current occupation?

1 MD/DO
2 NP
3 APRN
4 Dietitian
5 Other (please specify)

9. Years practicing pediatric gastroenterology?

1 GI fellow-years as a GI attending
2 2-5 years as a GI attending
3 6-10 years as a GI attending
4 11-20 years as a GI attending
5 21-30 years as a GI attending
6 31+ years as a GI attending

10. Years in pediatric gastroenterology?

1 0-1years
2 2-5 years
3 6-10 years
4 11-20 years
5 21-30 years
6 31+ years

C. Telemedicine Experience and Training

11. Previous telemedicine training (select all that apply):

a Colleagues

b. Medical literature
c. Formal in-person or virtual training
d. Meetings/conferences
e. Handouts/job aids only
f. Online webinars
g. No previous training
h. Other (please specify)

12. Knowledge level about telemedicine pre-COVID?

1. No knowledge at all
2. Somewhat knowledgeable
3. Knowledgeable
4. Very knowledgeable

13. Knowledge level about telemedicine currently?

1. No knowledge at all
2. Somewhat knowledgeable
3. Knowledgeable
4. Very knowledgeable

14. I use telemedicine currently for?

1. 0-25% of my patient volume
2. 26-50% of my patient volume
3. 51-75% of my patient volume
4. 76-100% of my patient volume

D. Current Use of Telehealth

15. Current Use of Telehealth?

a. Initial Visit
b. Follow up
c. Second opinion
d. Chronic disease care
e. E-consults/interprofessional communication

16. Technical Considerations with Telehealth?

a. Technical/equipment issues impact the visit
b. Software issues impact the visit
c. Sound issues impact the visit
d. Technical assistance is available during the visit

17. Experience with Telehealth?

a. I am proficient at using the equipment for telemedicine (TAM domain SE)
b. I have rich experiences on telemedicine (TAM domain SE)
c. I am able to use the telehealth equipment properly (TAM domain SE)

d. It is easy to learn to use the software and equipment for telemedicine (TAM domain PEU)
e. It is easy to perform my job with the EMR and telemedicine software (TAM domain PEU)

18. Patient Care?

a. Most visits I do can be accomplished by video visits (TAM domain ATU)
b. I can gather the correct information and easily record a patient's health condition into the EMR (TAM domain AMR)
c. Because of the precise record of the patients, it enables me to provide appropriate care for my patients (TAM domain AMR)
d. Telemedicine will positively affect patient quality of care and treatment plans (TAM domain PU)
e. I can conduct a thorough patient exam using telemedicine (TAM domain PU)
f. Video visits are more acceptable for established patients rather than new patients (TAM domain PU)
g. I believe that my patients will receive better care in person than via telehealth (TAM domain PU)
h. With telehealth I am able to be in contact with patients who seldom come the clinic (TAM domain AP)
i. With telehealth I am able to be in contact with patients who have transportation difficulties to come to visits (TAM domain AP)
j. Telehealth is efficient for diagnosing patients and scheduling (TAM domain PU)
k. I feel that there is a loss of personal contact with patients that results from telemedicine (TAM domain ATU)
l. Telehealth makes it possible to provide more comprehensive care service (TAM domain PU)

19. Licensure, Reimbursement?

a. Current Medicare/Medicaid reimbursement during the PHE is adequate for my level of participation (TAM domain PI)
b. I would like the temporary expansion of telehealth to become permanent to allow for better access of care (TAM domain PI)
c. I feel concern regarding liability issues with telemedicine (TAM domain PI)
d. I am concerned about state medical licensure issues with telemedicine (TAM domain PI)
e. I will care for more of my patients using telemedicine in my practice (TAM domain BUI)

20. I am interested in participating in future activities with the Telehealth for Pediatric GI Care Now (TPGCN) group (please check all that apply):

a. Research
b. Quality Improvement
c. None

Appendix 1b: Post-Webinar Survey

A. Telehealth for Pediatric GI Care Now (TPGCN) group

2. Would you like to participate in this research?

   1 Yes
   2 No

3. Your email address?

4. Did you attend the NASPGHAN Telehealth Webinar?

   1 Yes
   2 No

B. Survey Experience

5. Please answer the following questions about your overall webinar experience:

   a I found the webinar content intellectually challenging and stimulating (SEEQ)
   b I learned something that I consider valuable (SEEQ)
   c My interest in the subject has increased as a consequence of this webinar (SEEQ)
   d I understood the subject materials of the webinar (SEEQ)
   e As a result of this webinar, I anticipate that my telemedicine practice will change (SEEQ)
   f The breakout sessions were informative and enjoyable (SEEQ)
   g The open Q&A format addressed issues that were not covered in the sessions (SEEQ)

6. I would attend similar webinar based formats for interactive learning?

7. How did you participate in the didactics?

   a Technical lecture (6/10/20)
   b Billing/Documentation lecture (6/10/20)
   c Practice Management lecture (6/17/20)

8. Please share any feedback or suggestion for the didactics (optional).

9. How did you participate in the breakout sessions?

   a Technical breakout (6/10/20)
   b Billing/Documentation breakout (6/10/20)
   c Inpatient and Tele-education breakout (6/17/20)
   d Practice Management breakout (6/17/20)

10. Please share any feedback or suggestion on the breakout sessions (optional).

11. Based on content discussed in the webinar, which areas of telemedicine would you want to learn more about?

a. Virtual visits
b. Cross border coverage
c. New patient visit
d. Telephone Visits
e. E-consults/provider to provider communication
f. Other (please specify)

What other content areas would you like to learn about?

12. I am interested in participating in future Research activities with the Telehealth for Pediatric GI CareNow (TPGCN) group (please check all that apply):

    1. Research
    2. Quality Improvement
    3. Research and Quality Improvement
    4. None of the above

13. Please provide any additional comments, questions, or concerns (optional).

C. Survey Knowledge

14. Which of the following are acceptable ways to bill for a telehealth visit?

    1. Standard components including a physical examination and medical decision making
    2. Face-to-face time based billing
    3. Total visit time base billing including pre-visit planning and post-visit care.
    4. Any of the above may be acceptable depending on the policies of your institution/practice

15. Which of the following are acceptable components of a telehealth visual physical examination?

    1. No hepatosplenomegaly
    2. No palpable masses appreciated
    3. Abdomen non-distended
    4. All of the above

16. The CPT GT telehealth modifier is a standard for all telehealth visits regardless of insurance type.

    1. True
    2. False

17. Obtaining informed consent before conducting telemedicine (telehealth, telephone and e-visits) is:

    1. A requirement in many but not all states
    2. A condition of reimbursement by some insurance providers
    3. A and B
    4. None of the above

18. Three weeks after discharge from an inpatient hospitalization, a patient's father contacts his GI provider via the electronic health record secure patient portal. He asks a question about the appearance of his child's gastrostomy tube (GT) site and includes a photo of the GT site in the message. The GI provider reviews the image the next day and responds with recommendations for management of granulation tissue. The GI provider documents the care provided and charges for the visit as a:

1 Telehealth visit
2 Virtual Check-in
3 E-visit
4 Telephone visit

19. Knowledge level about telemedicine currently?

1 No knowledge at all
2 Somewhat knowledgeable
3 Knowledgeable
4 Very knowledgeable

20. I use telemedicine currently for?

1 0-25% of my patient volume
2 26-50% of my patient volume
3 51-75% of my patient volume
4 76-100% of my patient volume

21. Current Use of Telehealth?

a Initial Visit
b Follow up
c Second opinion
d Chronic disease care
e E-consults/interprofessional communication

22. Technical Considerations with Telehealth?

a Technical/equipment issues impact the visit
b Software issues impact the visit
c Sound issues impact the visit
d Technical assistance is available during the visit

23. Experience with Telehealth?

a I am proficient at using the equipment for telemedicine (TAM domain SE)
b I have rich experiences on telemedicine (TAM domain SE)
c I am able to use the telehealth equipment properly (TAM domain SE)
d It is easy to learn to use the software and equipment for telemedicine (TAM domain PEU)
e It is easy to perform my job with the EMR and telemedicine software (TAM domain PEU)

24. Patient Care?

a. Most visits I do can be accomplished by video visits (TAM domain ATU)
b. I can gather the correct information and easily record a patient’s health condition into the EMR (TAM domain AMR)
c. Because of the precise record of the patients, it enables me to provide appropriate care for my patients (TAM domain AMR)
d. Telemedicine will positively affect patient quality of care and treatment plans (TAM domain PU)
e. I can conduct a thorough patient exam using telemedicine (TAM domain PU)
f. Video visits are more acceptable for established patients rather than new patients (TAM domain PU)
g. I believe that my patients will receive better care in person than via telehealth (TAM domain PU)
h. With telehealth I am able to be in contact with patients who seldom come the clinic (TAM domain AP)
i. With telehealth I am able to be in contact with patients who have transportation difficulties to come to visits (TAM domain AP)
j. Telehealth is efficient for diagnosing patients and scheduling (TAM domain PU)
k. I feel that there is a loss of personal contact with patients that results from telemedicine (TAM domain ATU)
l. Telehealth makes it possible to provide more comprehensive care service (TAM domain PU)

25. Licensure, Reimbursement?

a. Current Medicare/Medicaid reimbursement during the PHE is adequate for my level of participation (TAM domain PI)
b. I would like the temporary expansion of telehealth to become permanent to allow for better access of care (TAM domain PI)
c. I feel concern regarding liability issues with telemedicine (TAM domain PI)
d. I am concerned about state medical licensure issues with telemedicine (TAM domain PI)
e. I will care for more of my patients using telemedicine in my practice (TAM domain BUI)

Appendix Table 1: Webinar Syllabus

	June 10, Session 1	June 17, Session 2
**Didactic**	**Technical Aspects (30min)**<br>• Telehealth Platforms<br>• Privacy and Security<br>• Common Problems<br>• Troubleshooting<br><br>**Documentation, Billing, Reimbursement (30 min)**<br>• Consent and HIPAA Requirements<br>• Physical Exam – Key Elements<br>• Coding with Telehealth Modifiers<br>• Billing by Time	**Practice Management (40 min)**<br>• Pre-COVID Telehealth Barriers<br>• Patient Selection<br>• Pre-, Intra-, Post-visit<br>• Professionalism, Provider Wellness<br>• Pros and cons of telehealth
**Breakouts**	**Technical aspects (60 min)**<br>• Case-Based Discussions, Polls, Q&A<br><br>**Documentation and billing (60 min)**<br>• Case-based discussion, Polls, Q&A	**Practice Management (60 min)**<br>• Didactic, Polls, Q&A<br><br>**In-Patient Care and Tele-Education (60 min)**<br>• Didactic, Polls, Q&A

Appendix Figure 1: Study CONSORT diagram

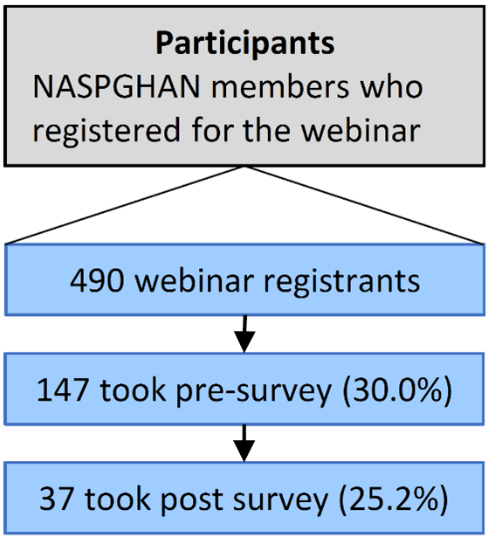

Appendix Figure 2: Venn diagram of Study Participants

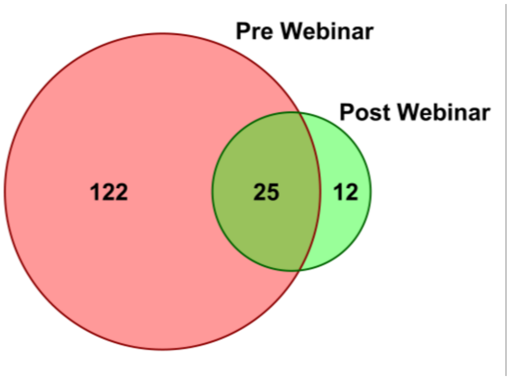

Appendix Figure 3: TAM survey domains[1-3]

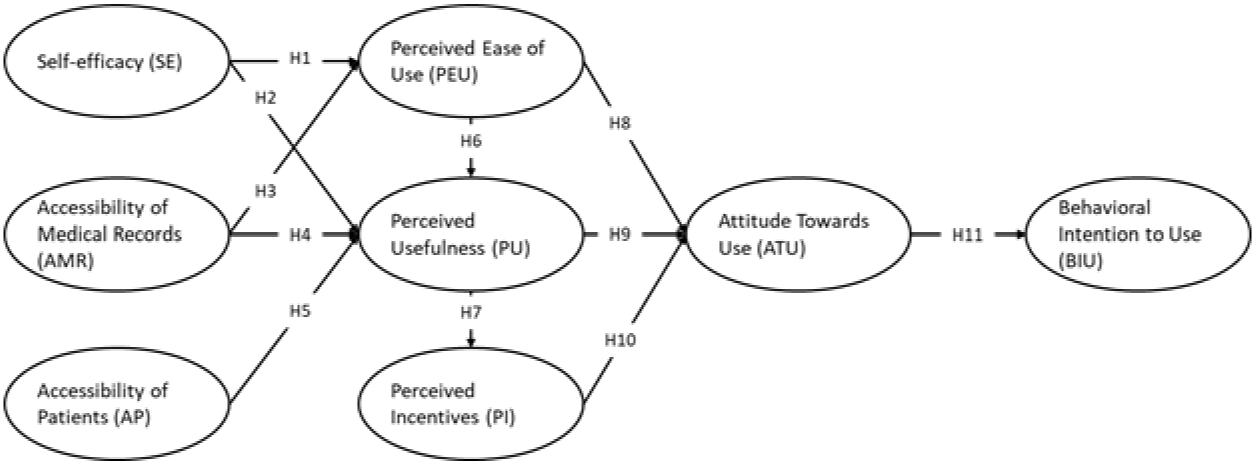

1. Davis FD. Perceived Usefulness, Perceived Ease of Use, and User Acceptance of Information Technology. MIS Quarterly 1989;13:319-340.
2. Ronnie HS, Christopher CD, Eugenia MWN. Analysis of the technology acceptance model in examining students' behavioural intention to use an e-portfolio system. Australasian Journal of Educational Technology 2011;27.
3. Sung Youl P. An Analysis of the Technology Acceptance Model in Understanding University Students' Behavioral Intention to Use e-Learning. Journal of Educational Technology & Society 2009;12:150-162.
